# Microbiota of human precolostrum and its potential role as a source of bacteria to the infant mouth

**DOI:** 10.1038/s41598-019-42514-1

**Published:** 2019-06-10

**Authors:** Lorena Ruiz, Rodrigo Bacigalupe, Cristina García-Carral, Alba Boix-Amoros, Héctor Argüello, Camilla Beatriz Silva, Maria de los Angeles Checa, Alex Mira, Juan M. Rodríguez

**Affiliations:** 1 IPLA-CSIC, Department of Microbiology and Biochemistry of Dairy Products, Institute of Dairy Products of Asturias, Villaviciosa, Spain; 2 Department of Nutrition and Food Science, Complutense University of Madrid, Avda. Puerta de Hierro, s/n, 28040 Madrid, Spain; 3 Centro Superior de Investigación en Salud Pública, Fundación FISABIO, Valencia, Spain; 4 Probisearch S.L., C/Santiago Grisolía, 2, 28760 Tres Cantos, Spain; 5 Grupo de Genómica y Mejora Animal, Departamento de Genética, Facultad de Veterinaria, Universidad de Córdoba, Córdoba, Spain; 6 Universidade de Uberaba, Uberaba, Brazil; 7 Centro de Salud Arrabal, 50015 Zaragoza, Spain

**Keywords:** Phylogenomics, Metagenomics, Bacteriology

## Abstract

Human milk represents a source of bacteria for the initial establishment of the oral (and gut) microbiomes in the breastfed infant, however, the origin of bacteria in human milk remains largely unknown. While some evidence points towards a possible endogenous enteromammary route, other authors have suggested that bacteria in human milk are contaminants from the skin or the breastfed infant mouth. In this work 16S rRNA sequencing and bacterial culturing and isolation was performed to analyze the microbiota on maternal precolostrum samples, collected from pregnant women before delivery, and on oral samples collected from the corresponding infants. The structure of both ecosystems demonstrated a high proportion of taxa consistently shared among ecosystems, *Streptococcus* spp. and *Staphylococcus* spp. being the most abundant. Whole genome sequencing on those isolates that, belonging to the same species, were isolated from both the maternal and infant samples in the same mother-infant pair, evidenced that in 8 out of 10 pairs both isolates were >99.9% identical at nucleotide level. The presence of typical oral bacteria in precolostrum before contact with the newborn indicates that they are not a contamination from the infant, and suggests that at least some oral bacteria reach the infant’s mouth through breastfeeding.

## Introduction

Maternal microbes exert important roles in determining the establishment and assembly of human microbiomes in early life and, therefore, have a notable impact on infant health in the short and long term^1–3^. Diverse factors including, among others, gestational age, delivery mode (vaginal delivery versus C-section), type of infant feeding (exclusive breastfeeding, infant formula, mixed feeding), time from birth to skin-to-skin contact, perinatal use of antibiotics or the health status of the mother (e.g., infections, diabetes, metabolic syndrome) significantly influence the process of acquisition and establishment of the infant microbiota^1,4–14^. Globally, such influences may have direct consequences on the infant metabolism, immunological and neuroendocrinological programming and ultimately on health outcomes later in life. In fact, those situations altering or disturbing the infant gut microbiota have been associated with an increased incidence of metabolic and inflammatory disorders later in adulthood^15–19^.

In this context, human milk seems to play a particularly relevant role in driving the establishment of the infant gut microbiota, attributed to its content in antimicrobial and immunological compounds, prebiotic substances (lactose, human milk oligosaccharides) and live microorganisms, among other biologically active components^20–23^. Indeed, breastfeeding has been demonstrated to represent a supply of bacteria for the infant gut and, the sharing of bacterial species and strains between maternal milk and the infant gut has been demonstrated in several occasions through both culture-independent and culture-dependent approaches^13,14,24–28^. While in the past, the presence of bacterial cells in human milk was considered the result of contamination originating from the infant’s oral cavity (streptococci) or the mother’s skin (staphylococci, propionibacteria)^29^, the detection of live bacterial cells and/or DNA from genera and species (including strict anaerobes) that are usually confined to the gut (*Bifidobacterium*, *Bacteroides*, *Parabacteroides, Blautia*, *Clostridium*, *Collinsella, Coprococcus*, *Faecalibacterium*, *Roseburia, Subdoligranulum*, …)^24,30^ has fueled a scientific debate on the origin of human milk bacteria^31^. In addition, human milk also contains many bacteria which are typical inhabitants of the oral cavity, ranging from *Streptococcus* to *Veillonella*, *Leptotrichia*, *Prevotella* or members of the TM7 phylum, whose presence in human milk has been generally attributed to contaminants originating in the infant mouth^32^. Therefore, although infant’s mouth and/or the maternal skin may provide some bacteria to the milk, this is not incompatible with the potential role of human milk as a source of bacteria to the infant^33,34^.

In this context, the aims of this work were first to analyze the microbiota of precolostrum, the biological fluid secreted by some women during late pregnancy, and thus before having had any contact with the infant’s mouth; and next, to determine the potential transfer of bacterial strains present in precolostrum at the end of pregnancy, to the infant mouth.

## Material and Methods

### Participating women and sample collection

A total of 17 mother-infant pairs were recruited for their participation in the study. To be eligible for participation, women had to meet the following criteria: (1) ≥18 year of age; (2) a healthy pregnancy ≥37 weeks; (3) secreting precolostrum; (4) intention to breastfeed their babies; and (5) normal body mass index (18.5 to 24.9 before pregnancy). Exclusion criteria included underlying conditions (including breast pain) and maternal use of antibiotics in the previous 30 days. The main characteristics of the recruited women are presented in Table 1.

**Table 1:** Demographic and clinical characteristics of the mother/infant pairs, that provided precolostrum and infant oral samples for this study.

Mother-infant pair code	Age (years)	Pregnancy number	Gestational age (weeks)	Infant age (days, at sampling)	Delivery mode	Use of antibiotics during pregnancy
1	35	Second	37	7	Vaginal	Yes (at week 21; 7 days)
2	35**	Second	37	6	Vaginal	No
3	32**	First	39	6	Vaginal	No
4	36	Second	37	7	Vaginal	No
5	45	First	40	7	Vaginal	No
6	31	First	39	5	Vaginal	No
7	33	First	39	5	Vaginal	No
8	41	Second	37	6	Vaginal	No
9	33	First	38	7	C-section	Yes (at week 14; 10 days)
10	34	First	37	6	Vaginal	No
11	32	First	38	5	Vaginal	No
12	34	First	40	5	Vaginal	No
16	31*	First	38	6	Vaginal	No
17	29	Second	41	7	C-section	No
18	33	First	39	6	Vaginal	No
19	30	First	40	7	Vaginal	No
20	29	First	38	7	Vaginal	No

*Include a pair of twins.

**Samples collected from these two mother-infant pairs could not be analysed through 16S metagenomic sequencing due to low quality of obtained DNA.

Precolostrum samples were collected at week 38–40 of pregnancy by manual expression using gloved hands. For this purpose, the mammary areola and nipple were first cleaned with prepackaged castile soap towelettes (Professional Disposables International, Inc.; Orangeburg, NY) and, later, soaked in chlorhexidine (Cristalmina, Salvat, Spain). The first drops were discarded and, subsequently, a fraction of ~500 μL was absorbed into a sterile cotton swab. Infant oral samples were collected 5–7 days after delivery by rubbing the infant gums with a sterile cotton swab. Samples were immediately placed in cold box (4 °C) and, within 1 hour, they were frozen and kept at −20 °C until processed.

All mothers participating in the study gave written informed consent to the protocol (reference 10/017E), which had been previously approved by the Ethical Committee of Clinical Research of Hospital Clínico San Carlos, Madrid (Spain). The study was carried out in accordance with the Declaration of Helsinki.

### Culture-dependent analysis

Both precolostrum and infant oral samples were retrieved from the cotton swabs through suspension into 1 ml of sterile peptone water and vigorous shaking. Then 50 μl of serial ten-fold dilutions of each sample were spread on Columbia Nadilixic Acid (BioMerieux) (CNA), MacConkey (BioMerieux) (MCK), and de Man, Rogosa and Sharpe (Oxoid) supplemented with 0.25% g L^−1^ L-cysteine hydrochloride (Sigma) (MRSc) agar plates. CNA and MCK plates were incubated aerobically at 37 °C for 2 days while MRSc plates were incubated anaerobically at 37 °C for 3 days, prior to colony picking. Representative colonies from each plate exhibiting different morphologies were stored in glycerol stocks and further identified by Matrix Assisted Laser Desorption Ionization-Time of Flight (MALDI-TOF) mass spectrometry using a Vitek-MS™ instrument (BioMérieux, Marcy l’Etoile, France). Briefly, a portion of a bacterial colony (~1 µL) was directly spotted onto a MALDI sample plate. Then, it was overlaid with 1 µL of a saturated solution of α-cyano-4-hydroxycinnamic acid in acetonitrile (28%) and allowed to dry at room temperature. For each isolate, a mean spectrum was constructed with at least 50 m/z spectra profiles and used for the identification by comparison with the spectra contained in the Myla database (Biomerieux). Identification was defined as a 99–100% match to the species-specific *m*/*z* values in the database.

In order to determine potential maternal-infant sharing of bacteria at the strain level, those isolates belonging to the same species and obtained from the precolostrum and infant oral samples of the same mother-infant pair were further genotyped by RAPD-PCR profiling following the procedure described by Martín and colleagues^26^ and whole genome sequencing as described below. Samples of mature milk and infant mouth from 3 additional mother-infant pairs collected 8 weeks after birth were also included in this part of the work. These were used as a kind of positive controls since potential sharing of strains between precolostrum and infant mouth was unknown at the beginning of this study while a high degree of strain sharing between mature milk and infant mouth was expected^14,33,34^.

### DNA extraction, amplification and 16S rRNA sequencing

Both precolostrum and infant oral samples were preserved and processed identically as previously described^35^. Briefly, suspended cells were collected by centrifugation at 13,000 ×*g* for 10 min at 4 °C and suspended in 0.5 mL TE50 (10 mM Tris-HCl, 50 mM EDTA, pH 8). Then, they were subjected to enzymatic lysis by adding 100 µL of a mixture containing lysozyme (Sigma-Aldrich), mutanolysin (Sigma-Aldrich) and lysostaphin (Sigma-Aldrich) as previously described^35^ and incubating the samples for 1 h at 37 °C. Immediately afterwards, physical disruption was conducted by 3 bead-beating cycles of 1 min each, on a FastPrep instrument (QBioGene, Irvine, CA, USA) by using 0.1 mm zirconia/silica beads (Sigma). Following cell lysis, DNA was purified from the sample by using the QIAmp DNA Mini Kit (Qiagen) according to the manufacturer’s instructions.

Extracted DNA was eluted in 22 µL nuclease-free water and stored at −20 °C until further analysis. A dual-barcoded 2-step PCR was conducted to amplify a fragment of the V3–V4 hypervariable region of the bacterial 16S ribosomal RNA (rRNA) gene; by using equimolar concentrations of the universal primers S-D- Bact-0341-b-S-17 (ACACTGACGACATGGTTCTACACCTACGGGNGGCWGCAG) and S-D-Bact-0785-a-A-21 (TACGGTAGCAGAGACTTGGTCTGACTACHVGGGTATCTAATCC) as previously described^36^, generating ~464 bp amplicons from the V3 to V4 hypervariable regions. Barcodes used for Illumina sequencing were appended to 3′ and 5′ terminal ends of PCR amplicons to allow separation of forward and reverse sequences. A bioanalyzer (2100 Bionalyzer, Agilent) was used to determine the concentration of every sample in the region of interest.

Two negative control blanks, which included no sample, were subject to all steps of the DNA extraction and purification procedure described above. The concentration of DNA in the two blank preparations was approximately 0.01 ng µl^−1^ while that obtained from the biological samples that were included in the 16S rRNA sequencing analysis was, at least, 159.9 ng µl^−1^. In addition, no amplification was detected from the blank samples after the first PCR and, as a consequence, the two blank controls were not submitted to sequencing.

Barcoded PCR products from all samples were pooled at approximately equal molar DNA concentrations and run on a preparative agarose gel. The correct sized band was excised, the DNA was purified and one aliquot of pooled, purified, barcoded DNA amplicons was sequenced on an Illumina MiSeq machine using a pair-ends 2 × 250 bp protocol (Illumina Inc., San Diego, CA, USA) at the Unidad de Genómica of the Fundación Parque Científico de Madrid (Spain). All sequences generated in this work are deposited in the Short Reads Archive under bioproject number PRJNA489791.

### Processing of 16S rDNA amplicon sequences

Raw sequencing reads of V3-V4 amplicons of the 16S rRNA gene amplicons were processed by using the version 1.9.1 of the Quantitative Insights Into Microbial Ecology (QIIME) software, using the open-reference OTU calling approach^37,38^. Briefly, reads were demultiplexed and primers and barcodes were trimmed using QIIME’s default settings. Following trimming, paired end sequence reads were merged with default QIIME settings and clustered into OTUs against the GreenGenes database (Version 13_8) by using the parallel uclust_ref method^39^. Reads with no hits in the reference sequence collection were clustered with *de novo* OTU method. Singletons and doubletons OTUs were filtered out, and only those OTUs representing more than 0.001% of the total filtered taxa were retained for diversity analyses^40^. For conducting statistical analyses at genus level, OTUs were collapsed into the corresponding taxonomic level by using the tax_glom functions in PhyloSeq^41^.

### Whole genome sequencing of mother-child paired isolates and comparative genomics

DNA from 10 pairs of bacterial isolates (each pair represented by one isolate from precolostrum and one isolate from the corresponding infant’s oral cavity) that appeared to be identical by MALDI-TOF and RAPD-PCR profiles was extracted by a combination of physical and chemical lysis as described above. The 20 isolates were sequenced in the same run with a Miseq Illumina platform using standard protocols from the manufacturer, with a 2 × 300 bp paired-ends kit.

Illumina reads were trimmed using Trimmomatic 0.36^42^, removing adapters, short reads and leading bases with a quality below Q3. Trimmed reads were assembled using SPAdes 3.10^43^ and contigs shorter than 1 Kb were discarded. Due to the risk of contamination in Illumina assemblies^44^, we searched for and filtered out contigs with coverage values and GC content that significantly deviated from those expected for our genomes.

In order to contextualize each pair of isolates among the known species genomic diversity, we selected 3 to 6 assembled genomes of the same or close related species from the NCBI database, depending on availability of fully-sequenced isolates. Average Nucleotide Identity (ANI), a measure of nucleotide-level genomic similarity between the genomes of two bacterial isolates, was estimated between all the pairs of isolates of each species using the ANI method from pyani (https://github.com/widdowquinn/pyani). Next, for each of these groups, we identified core genes using Roary with default parameters^45^ and core genome alignments were constructed, which were then used to build ML trees with FastTree^46^. Finally, assembly comparisons between pairs of isolates and alignment graphs were constructed using circoletto^47^.

### Statistical analysis

All the statistical analyses were performed in Rstudio (version 3.3.2.1). Microbiota profiles and study variable (Sample type) were included in the estimation of alpha diversity (Shannon, Chao1 and Simpson indexes) and beta diversity analysis with the Vegan R package^48^. Dissimilarities between pairs of samples were estimated with the Bray-Curtis dissimilarity index and Unifrac indexes^49^ and analyzed with Principal Coordinate Analysis (PCoA) in Vegan^50^. The Vegan *envfit* function was used to evaluate if the factor of study (Sample type) was associated to the PCoA ordinations by using permutational multivariate analysis of variance using distance matrices (adonis)^51^, which was employed to describe the strength and significance that a categorical factor has in determining variation of ecological distances. The significance of the fitted factor was estimated by using 999 permutations.

A minimum of 35,000 sequences per sample were used for standardizing the microbiota measures. Rarefaction curves indicated that the sequencing depth was enough since the samples reached the plateau phase (Supplementary Fig. 1). Thus for subsequent core-microbiota and statistical analysis of compositional data, relative abundance was estimated by total-sum scaling normalization. The extent of microbiota shared across all the analyzed mother-infant pairs was estimated as the overlap between the core microbiotas at each site (infant saliva and maternal precolostrum) at genus level. The core microbiota for each sample type was predicted as those genera present in ≥50% of the samples and presenting a mean relative abundance above 1% within the corresponding groups (infant saliva and maternal precolostrum), using the core function in the microbiome R package^52^. Sharing of bacterial taxa between maternal precolostrum and infant oral microbiota beyond the predicted core microbiotas was studied at OTU level within every mother-infant pair.

Differences in the relative abundances of specific taxa among the different sample types collected were analyzed statistically at phylum, family and genus levels, in those taxa exhibiting a mean relative abundance above 0.1%. Due to the lack of normality or homogenous variances in some of the variables analyzed the non-parametric U-Mann-Whitney test was used to asses taxa differentially represented in both sample types. All *p* values were corrected when appropriate by using the Benjamini-Hochberg false discovery rate (FDR) correction. Significance was established at a *p* < 0.05 threshold for all the statistical analysis conducted.

## Results

### Characteristics of the population

The study was conducted on samples collected from 17 healthy mothers (aged 29–45) and their corresponding infants, including a mother and a pair of twins. A sample of precolostrum was obtained from the pregnant mother at 38–40 weeks of pregnancy, before delivery, and a swab sample from the gums of the corresponding infant was obtained 5–7 days after birth. All infants were exclusively breastfed and received no specific medication. Only two mothers delivered by C-section and 12 of them were primiparous (Table 1).

### Analysis of microbiome community structure in precolostrum and infant oral samples

**Figure 1:**
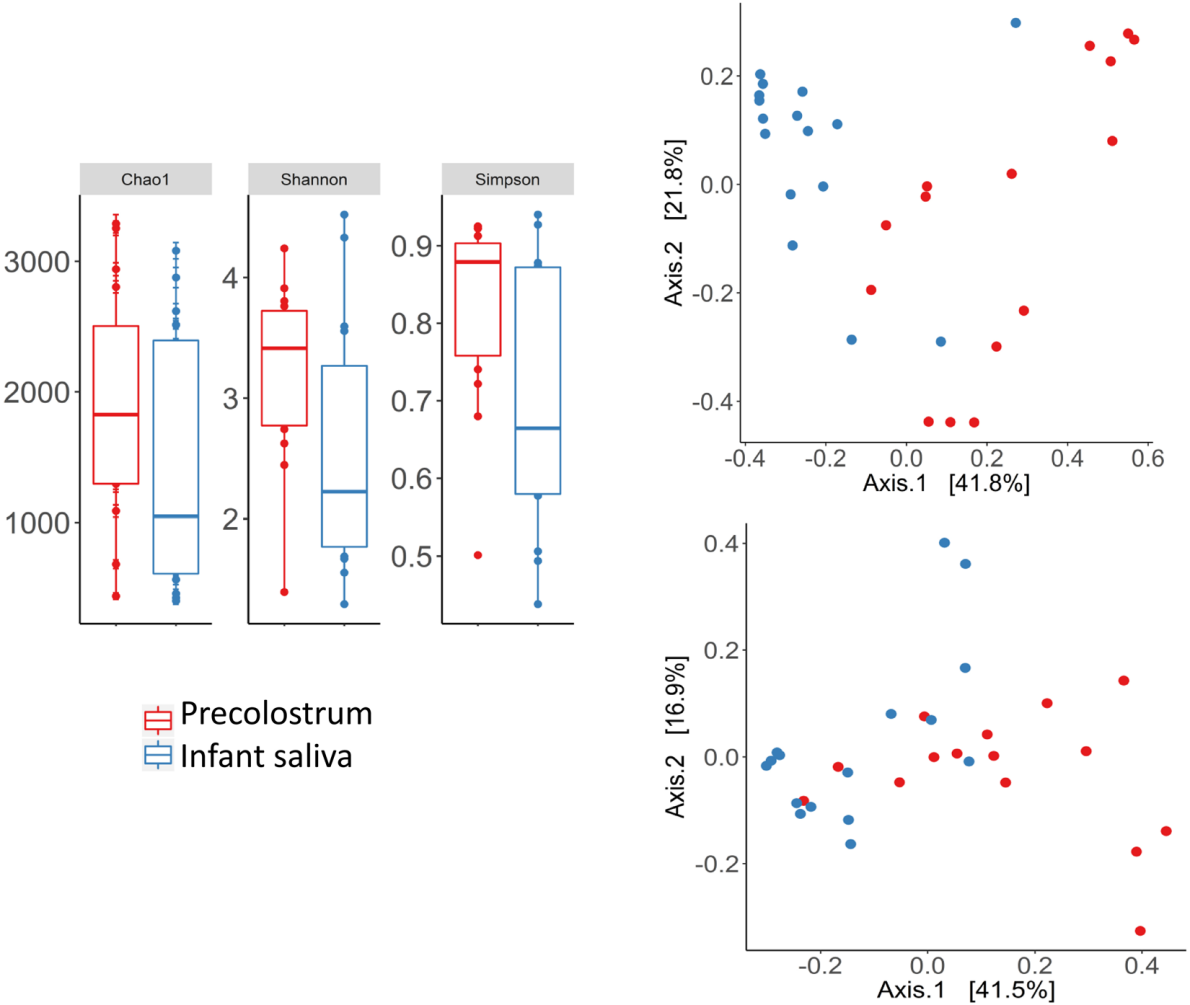
(**A**) Box plots of 3 different alpha diversity measures: the Chao I estimator, the Shannon index and Simpson index based on OTUs clustered at 97% similarity for precolostrum (red) and infant oral samples (blue). (**B**) Principal coordinates analysis of infant oral (blue) and maternal precolostrum (red) samples. Distances are based on the Bray-Curtis distances (upper right pannel), or weighted Unifrac distance metric (bottom right pannel), calculated using normalized data (PCoA based on unweighted Unifrac distance metrics provided very similar results to those calculated with weighted Unifrac).

16S rRNA gene taxonomic profiling was conducted in samples collected from 15 of the 17 participant mother-infant pairs, as for the remaining two sample pairs, the quantity and quality of DNA yield was below the threshold required for the sequencing service (Unidad de Genómica of the Fundación Parque Científico de Madrid). Overall, this analysis resulted in the generation of a total of 3,683,325 reads, representing on average 81,060 (±33,469 SD) reads per sample in the precolostrum samples and 72,504 (±35,874 SD) reads per sample in the infant oral samples. After filtering out singletons and OTUs with low relative abundance, 1,877 OTUs were retained for diversity analyses. Sample richness Chao1 index, diversity Shannon index and evenness Simpson index were estimated across the two sample groups (precolostrum and infant mouth). Overall, precolostrum samples displayed higher alpha diversity values than the infant oral samples, as determined through Shannon and Simpson indexes (U Mann-Whitney *p* = 0.037 and *p* = 0.040, respectively). (Fig. 1A).

**Figure 2:**
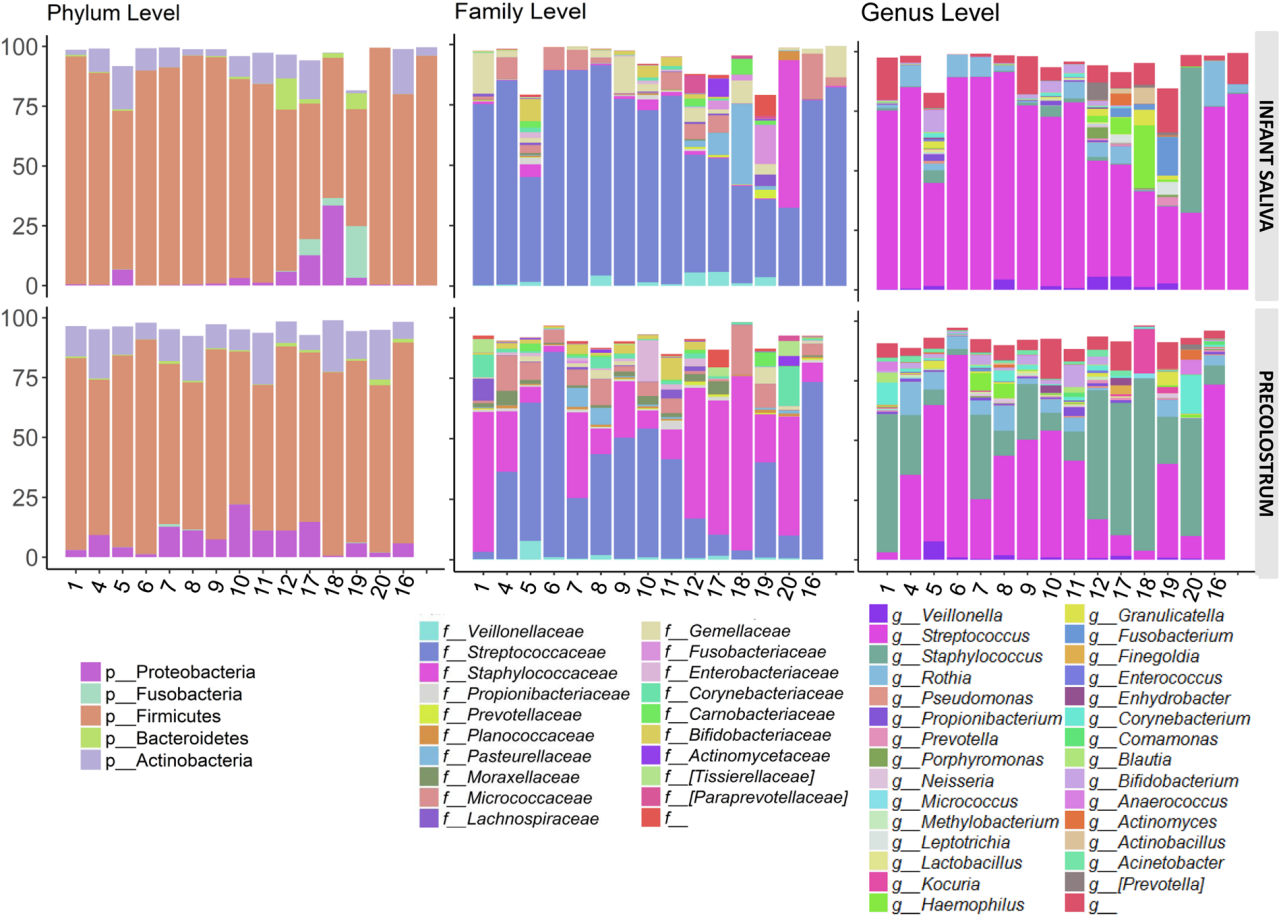
Relative abundances of most abundant taxa at phyla (left), family (middle) and genus (right) levels in both infant oral (upper pannels) and maternal precolostrum (bottom pannels) samples are represented.

In addition, a global principal coordinate analysis with all the available data evidenced that the type of sample (precolostrum *vs* infant mouth) significantly influenced samples ordination based both on Bray-Curtis dissimilarities and weighted and unweighted Unifrac distances (adonis: R^2^ = 0.27, *p* = 0.001; R^2^ = 0.18, *p* = 0.001; and R^2^ = 0.06, *p* = 0.013, respectively) (Fig. 1B). There was no separation between primiparous, delivery type or mother-infant pairs (data not shown). After filtering low abundance groups (mean relative abundance <0.001%), 21 phyla, 137 families and 244 genera were retrieved for further statistical analyses. From those groups, the dominant phylum across all the sample types analyzed was Firmicutes (mean relative abundance 77.22% ± 13.44), followed by Actinobacteria (mean relative abundance 10.51% ± 6.58) and Proteobacteria (mean relative abundance 6.15 ± 7.48). Of note, these phyla were detected in all the samples included in the study, accounting for more than 90% of the OTUs detected. At family and genus levels, precolostrum samples were dominated by *Streptococcaceae* [*Streptococcus*] and *Staphylococcaceae* [*Staphylococcus*], followed by groups typically associated with the oral cavity or skin including *Micrococcaceae* [*Rothia* and *Kocuria*], *Corynebacteriaceae* [*Corynebacterium*] or *Veilonellaceae* [*Veillonella*], in addition to groups typically associated with the intestinal ecosystem like *Bifidobacteriaceae* [*Bifidobacterium*]. Infant oral samples shared some of the dominant groups with precolostrum samples, as they were dominated by *Streptococcus* and *Staphylococcus*, followed by other groups typically associated with either the oral and nasopharingeal cavity (*Rothia, Haemophilus*, *Gemellaceae, Veillonella* or *Fusobacterium*) or even with the gut ecosystem (*Bifidobacterium*, *Veillonella*) (Fig. 2).

### Analysis of taxa shared or differentially represented in precolostrum and infant oral samples

Focusing on the differences and similarities of specific taxa between maternal precolostrum and infant oral samples at phylum level, Firmicutes were the dominant taxa in both sample types, followed by Proteobacteria and Actinobacteria, which were detected across all the samples analyzed. Bacteroidetes and Fusobacteria were only detected in a few of the infant oral samples (Fig. 2) although no statistically significant differences were detected for any of the phyla. When analyzed at the family and genus levels, the comparison between precolostrum and infant oral samples identified 11 families and 6 genera displaying statistically significant differences (FDR-value < 0.1, Table 2). Among them, significant differences were found in the top most abundant genera*: Staphylococcus*, which appeared overrepresented in precolostrum samples (FDR < 0.05); and *Streptococcus*, which appeared overrepresented in infant oral samples (FDR < 0.1). In addition, the most notable differences were a higher abundance of *Kocuria, Acinetobacter, Delftia*, and *Corynebacterium* in the precolostrum samples.

**Table 2:** Taxonomic groups displaying statistically significant differences between infant salivary and maternal precolostrum microbiotas.

Genus	Precolostrum		Saliva		FDR corrected *p*-value
	Median	IQR	Median	IQR	
*Staphylococcus*	23.350	45.96	0.550	1.29	0.001
*Delftia*	0.070	0.12	0.001	0.06	0.001
*Acinetobacter*	0.999	1.70	0.021	0.07	0.013
*Corynebacterium*	0.930	1.357	0.103	0.55	0.018
*Kocuria*	0.142	0.525	0.001	0.009	0.018
*Streptococcus*	39.258	43.71	75.917	39.705	0.098

Relative abundance data is presented in percentage, and p-values have been adjusted applying the Benjamini-Hochberg false discovery rate (FDR) correction.

**Figure 3:**
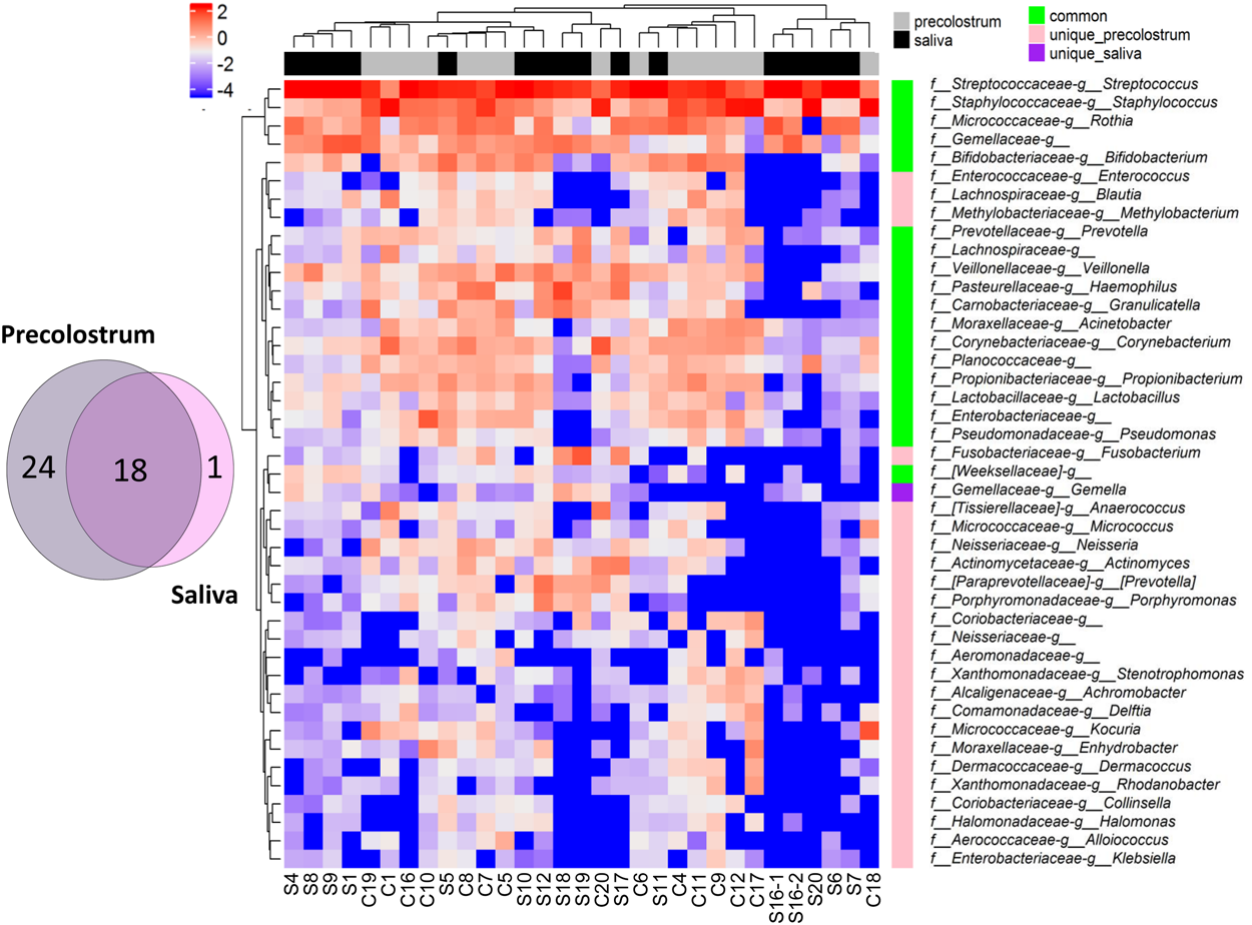
(**A**) Venn Diagram representing the overlap between the infant oral and the maternal precolostrum core microbiotas estimated at genus level. Core microbiotas were defined as taxa present at a relative abundance above 1% in at least 50% of the samples of the corresponding group. (**B**) Heatmap representing log2 relative abundances of the 43 genus comprising both precolostrum and infant oral core microbiotas. Samples and taxa clustering were performed on Euclidean distances using complete linkage.

A major aim of this work was to identify the taxa shared between the maternal precolostrum and the infant oral ecosystems. For this purpose, the core microbiomes estimated for each sample type were compared to determine the extent of overlapping among them and to discern the composition of a putative shared core microbiota, consistently shared between maternal precolostrum and infant mouth in most mother-infant pairs. A core group of 18 genera was found to be shared between both sample types in more than 50% of the mother-infant pairs included in the study (Fig. 3), which include typical taxa of the nasopharyngeal cavity, like *Haemophilus, Acinetobacter*, members of the Gemellaceae family*, Streptococcus, Granulicaella* and *Rothia*, in addition to a number of genera frequently detected in maternal milk samples, such as *Staphylococcus, Propionibacteria* and *Corynebacterium*. Notably, other bacterial groups typically associated with the gut ecosystem, including *Bifidobacterium, Veillonella*, and *Prevotella* (the latter two also common oral inhabitants), were also detected as part of the common shared core between maternal precolostrum and infant mouth, despite of their low relative abundance in both ecosystems. In order to further characterize the taxa shared between maternal precolostrum and linked infant’s mouth, we also analyzed the OTUs within each mother-infant cluster (Supplementary Tables 1 and 2). The extent of OTU sharing within each pair ranged between 19.2 to 65.38% of the detected OTUs, and twelve of the pairs shared more than 45% of the OTUs. Remarkably, 26 taxonomic groups were detected to be shared between maternal precolostrum and infant oral ecosystem in at least 5 of the analyzed pairs. Among them, the most frequently shared OTUs included *Streptococcus* and *Staphylococcus* members, which were also the dominant groups in the corresponding samples types. In addition, other abundant OTUs not included within predicted abundant core groups, such as *Bifidobacterium, Micrococcus, Blautia* or *Actinomyces*, were also shared in a significant proportion of the analyzed pairs.

### Culture-dependent analysis

**Figure 4:**
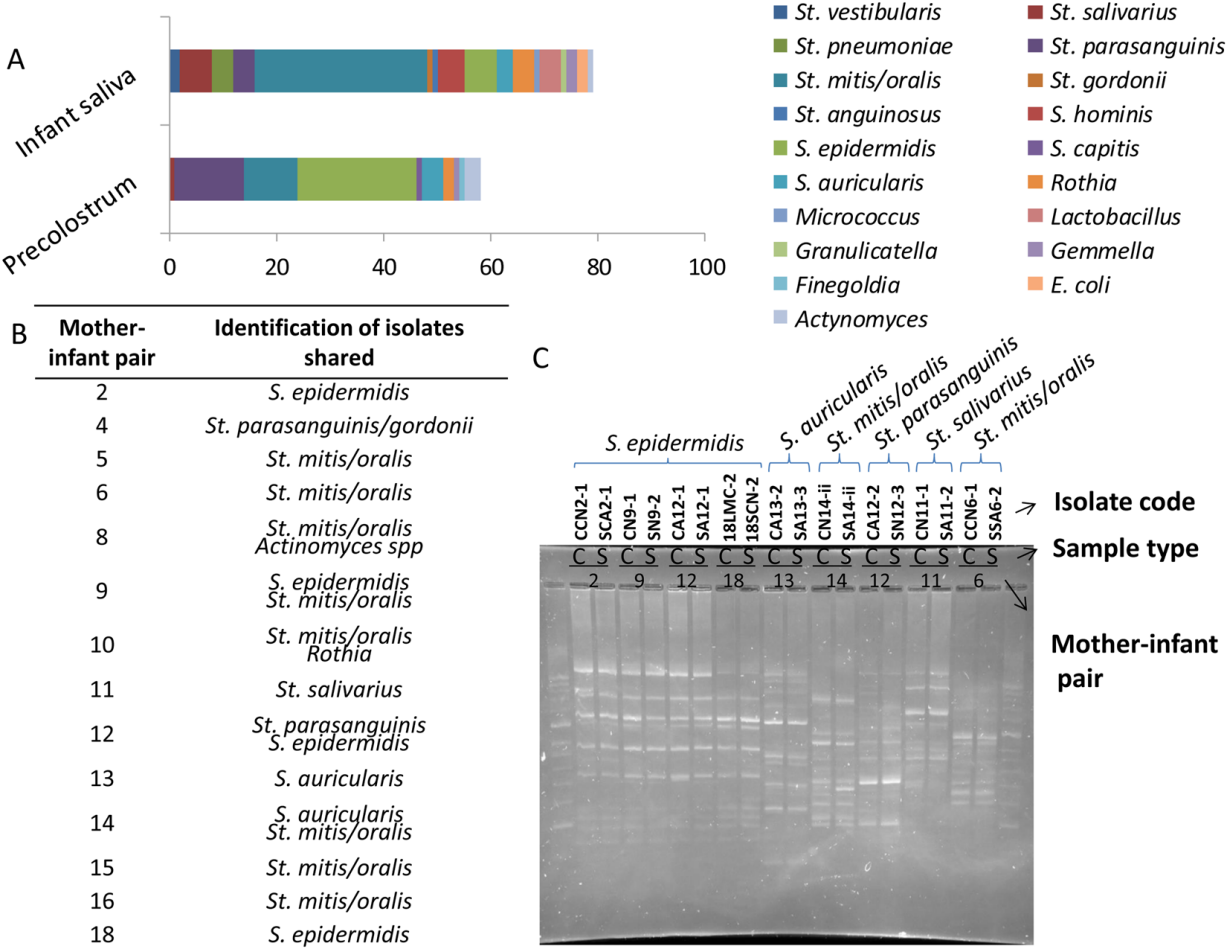
(**A**) Histogram representing the composition of the collection of isolates recovered from culturing infant oral and precolostrum samples. Identifications presented were performed through MALDI-TOF analysis. (**B**) Table summarising the mother-infant pairs in which bacteria sharing identical identification were recovered from both the precolostrum and the infant oral sample. (**C**) RAPD-PCR genotype profiling of those isolates that, belonging to the same genus/species were isolated from both samples from the same mother-infant pair and showed similar genotyping profile.

In combination to culture-independent metataxonomic analysis, an aliquot of each sample was used for culture-based analysis. The total bacterial load recovered from each sample ranged from 10^2^ to 10^4^ colony formation units per ml and a total number of 160 different colonies (58 from precolostrum samples and 79 from infant oral samples) were recovered, stocked and identified through MALDI-TOF (Fig. 4). The identification of bacteria recovered through culturing was consistent with the microbiota profiles previously described, with a higher number of *Streptococcus* and *Staphylococcus* isolates recovered from the oral and the precolostrum samples, respectively. In addition, it is also worth remarking that several isolates belonging to typical oral bacteria including *Rothia, Gemella* and *Actinomyces* species could be recovered from the infant mouth samples. Surprisingly, although most infants participating in the study were born vaginally, in this study a low proportion of lactobacilli species could be recovered through culturing from the infant oral samples, as opposed to previous reports based on culture-independent analysis^53^. Overall, in 11 of the analyzed mother-infant pairs, at least one isolate belonging to the same genus/species was recovered from both the precolostrum and the respective infant oral sample; it was also observed in 2 out of the 3 mature milk-infant mouth pairs (Fig. 4B). RAPD-PCR profiling revealed that in 9 cases, the isolates recovered from both sample types within the same pair could not be distinguished by RAPD-PCR genotype profiling. These isolates included four pairs classified as *Streptococcus epidermidis*, one pair as *Staphylococcus auricularis*, two pairs as *Streptococcus mitis/oralis*, one pair of *Streptococcus salivarius* and one pair as *Streptococcus parasanguinis*. Subsequently, such pairs of isolates were whole genome sequenced and genomic analyses were performed in order to confirm whether they were related strains. Only in one of the pairs (pair 8), *Actinomyces odontolyticus* were recovered from both the maternal and infant sample, and these strains were also subjected to whole genome sequencing despite they did not share RAPD-PCR profiles.

### Comparative genomics of matched pairs of isolated strains

**Figure 5:**
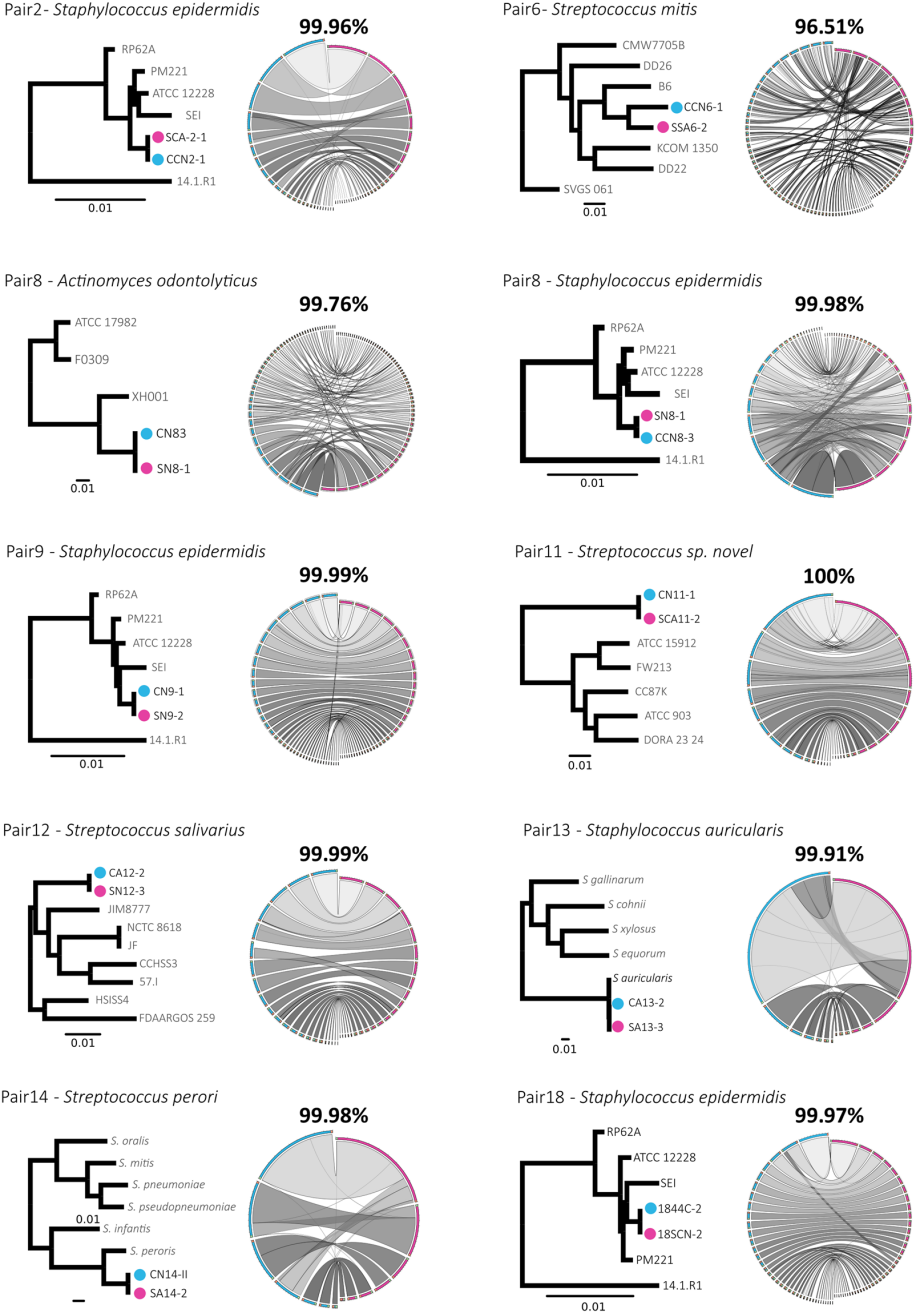
Whole genome sequencing comparison of mother-infant paired isolates. The genomes of each pair of isolates and 3–6 additional available genomes of the same species were compared by a phylogenomic tree constructed based on all shared genes. The blue and red dots indicate isolates from the precolostrum and oral samples of the baby, respectively. The average values of similarity (average nucleotide identity values) of the genomes for each mother-child pair are shown. Sinteny (conservation in gene order) is shown by lines that join the same fragments.

All isolates were sequenced at >50x coverage, facilitating the assembly of high-quality genomes. For each pair of isolates, a phylogenomic tree based on shared genes was constructed including 3 to 6 genomes from the same or closely related species, depending on availability in public repositories (Fig. 5, with precolostrum and infant oral isolates marked in blue and red, respectively). Next, we computed the Average Nucleotide Identity (ANI) values for each pair of isolates, a measure of DNA relatedness based on genomic similarity. Genomic synteny (i.e. conservation of gene order in the genome) is represented by lines joining homologous regions in the paired genomes. Notably, the pair 11 corresponds to a potential new streptococcal species, as evidenced by an ANI value to any described species well below the consensus of 95%, which is considered the species threshold^54^. In one of the 10 cases analyzed (pair 6), the two strains (belonging to *Streptococcus mitis*) appeared to be clearly different and in another case (the isolates pair 8, corresponding to *Actinomyces odontolyticus*) the two genomes showed an ANI of 99.76%. The other 8 pairs of analyzed genomes had a DNA identity over 99.9%, and in several instances, like pairs 9, 11 and 12, the genomes displayed a perfect synteny, strongly indicating that they correspond exactly to the same clone. It is also important to underline that the degree of phylogenomic similarity between the paired isolates is much higher than between any other available genome in public databases. Thus, these data indicate that the clones present in precolostrum appear a week later in the oral cavity of the corresponding infant, suggesting direct transmission from mothers to infants.

## Discussion

In this study, the microbiota of precolostrum and infant oral swabs obtained from 15 mother-infant pairs were analyzed and compared using a combination of 16S rRNA sequencing, bacterial isolation, whole genome sequencing and comparative genomics (WGS) of those isolates that seemed to be shared by both types of samples within the same mother-infant pair. These analyses show that precolostrum, secreted by women at the end of pregnancy, contains a bacterial profile very similar to that encountered in mature milk, which is dominated by *Staphylococcus* species, but including also species of bacterial groups typically found in the oral cavity (such as *Streptococcus*, *Fusobacterium, Veillonella* or *Porphyromonas*), in accordance with previous studies on mature human milk^30,32,55,56^. Notably, precolostrum samples from our study had not been in contact with the infant, thus ruling out the possibility that the bacterial presence in human milk might exclusively originate as contamination from either the skin and/or infant oral cavity. In addition, in some of the analyzed mother-infant pairs, our study isolated identical strains from precolostrum and from the mouth of the respective infant, a finding that suggests that human milk may have a significant impact in the initial establishment of the infant oral microbiota. It must be highlighted that, for many of the recruited women, this was their first pregnancy and therefore presence of oral bacteria in precolostrum cannot be explained on the basis of oral contamination from a previous child through breast suckling.

The initial assembly of the infant microbiomes in early life has been recognized as a key factor impacting infant health in the short and long terms and thus, understanding the forces driving its establishment has received increasing scientific attention in recent years^6,28^. Among the factors influencing such process, maternal microbiomes and, particularly, feeding mode are recognized as some of the major forces shaping the infant gut microbiome. In fact, breastfeeding is known to provide the infant a vast array of bioactive compounds including, among others, human milk oligosaccharides, which can act as prebiotic substrates for specific commensal microorganisms, as well as a set of commensal microorganisms to initially colonize the infant gut^21^. However, until recently, intra-mammary human milk was assumed to be a sterile fluid under physiological conditions and thus, the presence of bacteria in human milk has remained controversial. On the one hand, the common presence of staphylococci, streptococci or corynebacteria in expressed human milk was explained as the result of contamination arising from either the skin (staphylococci, corynebacteria, propionibacteria) or the infant’s mouth (streptococci)^57,58^. In the last case, ultrasound imaging of milk ejection revealed a certain degree of milk flow back into the mammary ducts during suckling, suggesting that bacteria from the infant’s oral cavity may contaminate milk^32,59^. However, the detection of live bacterial cells and/or DNA from gut-related facultative and strict anaerobe species prompted to hypothesize the existence of an endogenous entero-mammary pathway during late pregnancy and lactation, likely mediated through complex interactions between bacteria, immune cells and epithelial cells^60^. Although the existence or not of such physiological and selective translocation has been controversial and remains as a subject of debate^24,31,61,62^, subsequent work provided evidence in support of such hypothesis^63–69^. More recently, two lactic acid bacteria strains (*Lactococcus lactis* MG1614 and *Lactobacillus salivarius* PS2) were transformed with a plasmid containing the *lux* genes and orally administered to pregnant mice. The murine model allowed the visualization, isolation, and PCR detection of the transformed bacteria in different body locations, including mammary tissue and milk, reinforcing the hypothesis that physiological translocation of maternal bacteria during pregnancy and lactation may contribute to the composition of the mammary and milk microbiota^70^.

Many transient physiological changes occur during pregnancy and lactation, which may favor an increased bacterial translocation during late pregnancy and early lactation. For instance, hormonal action induces relevant oral changes during pregnancy, affecting the pH and microbiota composition; the gums become hyperemic and edematous and tend to bleed and, under such circumstances, the transfer of some pathogenic bacterial species and inflammatory compounds to the bloodstream have been associated with the onset of premature birth^71–73^. In this context, although coagulase-negative staphylococci and streptococci (salivarius and mitis groups) have been traditionally associated to the skin and oral cavity, respectively, they are widespread in most, if not all, human mucosal surfaces, reaching high concentrations in the mucosal surfaces of the digestive and genitourinary tracts. Therefore, these maternal mucosal sites may, thus, represent possible origins for bacterial translocation to precolostrum and breastmilk during the end of pregnancy in healthy hosts^33^.

Our data show that most bacterial genera are shared between precolostrum and the oral cavity of the neonate, further supporting that breastmilk could be one of the first microbial sources inoculating the oral cavity during the first days of life. Similarly, initial oral colonization may arise from different sources, including amniotic fluid swallowing, the vaginal environment and precolostrum, colostrum and human milk intake. It has already been shown that buccal administration of human colostrum has an impact on the oral microbiome of very low birth weight premature infants^74^. In addition, breastfeeding has recently been found the most important factor influencing oral microbiome colonization, having a long-term impact on bacterial composition that extends to 7 years of age^75^. In the present work, the mother-infant sharing of microorganisms at strain level was unequivocally demonstrated by means of a culture-dependent and whole comparative genomics approach. Indeed, a recent study had already demonstrated the existence of a high degree of strains sharing between maternal microbiomes at different body sites, and infant oral and gut microbiomes, through a metagenome approach^28^. However, to our knowledge, our work is the first to study human precolostrum as a potential source of microbes for the infant mouth, and to demonstrate through a combination of culture-independent and culture-dependent approach, the sharing of strains between both ecosystems in healthy mother-infant pairs. While the colonization process of the infant gut microbiome has been studied extensively, the origin of the oral microbiota and the factors dictating initial oral microbiota development are far from elucidated and deserve research attention due to its relevant implications for human health. Although the human oral microbiome is still widely unknown, *Streptococcus* species (e.g., *Streptococcus salivarius*) are considered as pioneer microorganisms that predominate in the initial oral microbial load^75–80^, and stimulate changes in the oral cavity (such as production of extracellular polymers) that favor the attachment and growth of subsequent species^81^. Thus, the right, timely exposure to the prebiotic and probiotic components of breastmilk could be vital for oral microbiota development. In cohort studies where oral samples have been analyzed from birth through several years, other factors influencing microbiota development were delivery mode (with a short-term impact) and antibiotic treatment during the first 2 years of life, which appeared to have a long-term impact in bacterial composition^75^.

The study faced some limitations, such as the limited number of recruited women, a fact that may be explained by the nature of the type of sample studied (precolostrum), which is only available in a small subset of pregnant women and, usually, at small amounts. Besides, we acknowledge that sequencing low biomass samples, such as those analyzed in this study, is currently a challenging process that may result in skew results due to possible introduction of contaminants during the DNA manipulation and sequencing process. In this regards, no amplification was detected in the first PCR round from two blank controls submitted to the same extraction procedure that the biological samples analyzed in this work. Nevertheless, this study provides, to our knowledge, the first analysis on the microbiological composition of human precolostrum secreted by pregnant women during their last third of pregnancy. Besides, our results suggest that this fluid already harbors a microbiota quite similar to the mature milk microbiota, and has an important role in initiating the infant oral microbiota establishment. Further studies are required in order to discern the pathways by which typical oral bacteria reach the mammary gland before any contact with the infant mouth.

## Supplementary information

Supplementary Material

## Data Availability

Sequences generated in this study have been deposited in the NCBI public repository under Accession Number #PRJNA489791.
