# Supplementary material for: Microbiota of human precolostrum and its potential role as a source of bacteria to the infant mouth
Source: Sci Rep. 2019 Jun 10;9:8435. doi: 10.1038/s41598-019-42514-1 (PMC6557856; doi:10.1038/s41598-019-42514-1)

**Microbiota of human precolostrum and its potential role as a source of bacteria to the infant** **mouth**

Lorena Ruiz^1,2§*^, Alba Boix-Amoros^3§^, Cristina García-Carral^2,4§^, Rodrigo Bacigalupe^3^, Héctor Argüello^5^, Camilla Beatriz Silva^2,6^, Maria de los Angeles Checa^7^, Alex Mira^3*^, Juan M. Rodriguez^2*^

^1^IPLA-CSIC, Department of Microbiology and Biochemistry of Dairy Products, Institute of Dairy Products of Asturias, Villaviciosa, España

^2^Department of Nutrition and Food Science, Complutense University of Madrid, Avda. Puerta de Hierro, s/n, 28040 Madrid, Spain

^3^Centro Superior de Investigación en Salud Pública, Fundación FISABIO, Valencia. Spain

^4^Probisearch S.L., C/ Santiago Grisolía, 2, 28760 Tres Cantos, Spain

^5^Grupo de Genómica y Mejora Animal, Departamento de Genética, Facultad de Veterinaria, Universidad de Córdoba, Córdoba, Spain

^6^Universidade de Uberaba, Brazil

^7^Centro de Salud Arrabal, 50015 Zaragoza, Spain

^§^These authors contributed equally.

*Corresponding authors

Supplementary Table 1. Summary of the number of OTUs detected in either one or the two samples analyzed within each mother-infant pair.

| **Mother-infant**  **pair code** | **1** | **4** | **6** | **7** | **8** | **9** | **10** | **11** | **12** | **16-1** | **16-2** | **17** | **18** | **19** | **20** |
| --- | --- | --- | --- | --- | --- | --- | --- | --- | --- | --- | --- | --- | --- | --- | --- |
| Associated to precolostrum | 329 | 106 | 67 | 100 | 111 | 71 | 140 | 106 | 116 | 102 | 102 | 70 | 80 | 165 | 143 |
| Shared in both ecosystems | 173 | 208 | 155 | 138 | 342 | 262 | 523 | 433 | 226 | 182 | 149 | 77 | 43 | 247 | 193 |
| Associated to  infant mouth | 68 | 59 | 47 | 50 | 75 | 125 | 137 | 121 | 150 | 69 | 56 | 106 | 101 | 132 | 71 |
| sum otus within pair | 570 | 373 | 269 | 288 | 528 | 458 | 800 | 660 | 492 | 353 | 307 | 253 | 224 | 544 | 407 |
| % otus shared | 30.4 | 55.8 | 57.6 | 47.9 | 64.8 | 57.2 | **65.4** | 65.6 | 45.9 | 51.6 | 48.5 | 30.4 | **19.2** | 45.4 | 47.4 |

Supplementary Table 2. OTUs classified up to the genus level, shared between maternal precolostrum and infant oral microbiotas in at least 5 of the mother-infant pairs

|  | **Taxonomy** | | | | | | **No. OTUs shared** |  |
| --- | --- | --- | --- | --- | --- | --- | --- | --- |
| p__Actinobacteria | | c__Actinobacteria | o__Actinomycetales | f__Propionibacteriaceae | g__Propionibacterium | 6 | | |
| p__Actinobacteria | | c__Actinobacteria | o__Bifidobacteriales | f__Bifidobacteriaceae | g__Bifidobacterium | 15 | | |
| p__Actinobacteria | | c__Coriobacteriia | o__Coriobacteriales | f__Coriobacteriaceae | g__Collinsella | 1 | | |
| p__Actinobacteria | | c__Actinobacteria | o__Actinomycetales | f__Micrococcaceae | g__Micrococcus | 1 | | |
| p__Actinobacteria | | c__Actinobacteria | o__Actinomycetales | f__Actinomycetaceae | g__Actinomyces | 1 | | |
| p__Actinobacteria | | c__Actinobacteria | o__Actinomycetales | f__Corynebacteriaceae | g__Corynebacterium | 5 | | |
| p__Bacteroidetes | | c__Bacteroidia | o__Bacteroidales | f__[Paraprevotellaceae] | g__[Prevotella] | 1 | | |
| p__Firmicutes | | c__Bacilli | o__Lactobacillales | f__Streptococcaceae | g__Streptococcus | 101 | | |
| p__Firmicutes | | c__Bacilli | o__Bacillales | f__Staphylococcaceae | g__Staphylococcus | 23 | | |
| p__Actinobacteria | | c__Actinobacteria | o__Actinomycetales | f__Micrococcaceae | g__Rothia | 13 | | |
| p__Actinobacteria | | c__Actinobacteria | o__Actinomycetales | f__Micrococcaceae | g__Micrococcus | 2 | | |
| p__Actinobacteria | | c__Actinobacteria | o__Actinomycetales | f__Micrococcaceae | g__Kocuria | 2 | | |
| p__Firmicutes | | c__Clostridia | o__Clostridiales | f__Veillonellaceae | g__Veillonella | 10 | | |
| p__Firmicutes | | c__Clostridia | o__Clostridiales | f__Lachnospiraceae | g__Blautia | 1 | | |
| p__Firmicutes | | c__Bacilli | o__Lactobacillales | f__Carnobacteriaceae | g__Granulicatella | 2 | | |
| p__Firmicutes | | c__Clostridia | o__Clostridiales | f__[Tissierellaceae] | g__Finegoldia | 1 | | |
| p__Firmicutes | | c__Bacilli | o__Lactobacillales | f__Lactobacillaceae | g__Lactobacillus | 6 | | |
| p__Firmicutes | | c__Clostridia | o__Clostridiales | f__[Tissierellaceae] | g__Anaerococcus | 1 | | |
| p__Firmicutes | | c__Clostridia | o__Clostridiales | f__Ruminococcaceae | g__Ruminococcus | 2 | | |
| p__Proteobacteria | | c__Gammaproteobacteria | o__Pasteurellales | f__Pasteurellaceae | g__Haemophilus | 1 | | |
| p__Proteobacteria | | c__Betaproteobacteria | o__Neisseriales | f__Neisseriaceae | g__Neisseria | 1 | | |
| p__Proteobacteria | | c__Gammaproteobacteria | o__Enterobacteriales | f__Enterobacteriaceae | g__Klebsiella | 1 | | |
| p__Proteobacteria | | c__Gammaproteobacteria | o__Enterobacteriales | f__Enterobacteriaceae | g__Enterococcus | 2 | | |
| p__Proteobacteria | | c__Gammaproteobacteria | o__Pseudomonadales | f__Moraxellaceae | g__Enhydrobacter | 1 | | |
| p__Proteobacteria | | c__Gammaproteobacteria | o__Pseudomonadales | f__Moraxellaceae | g__Acinetobacter | 3 | | |
| p__Proteobacteria | | c__Gammaproteobacteria | o__Xanthomonadales | f__Xanthomonadaceae | g__Rhodanobacter | 1 | | |

Supplementary Figure 1. Rarefaction curves generated for the 16S rRNA sequences obtained from the samples by using the Chao1 index (A) and Shannon index (B).


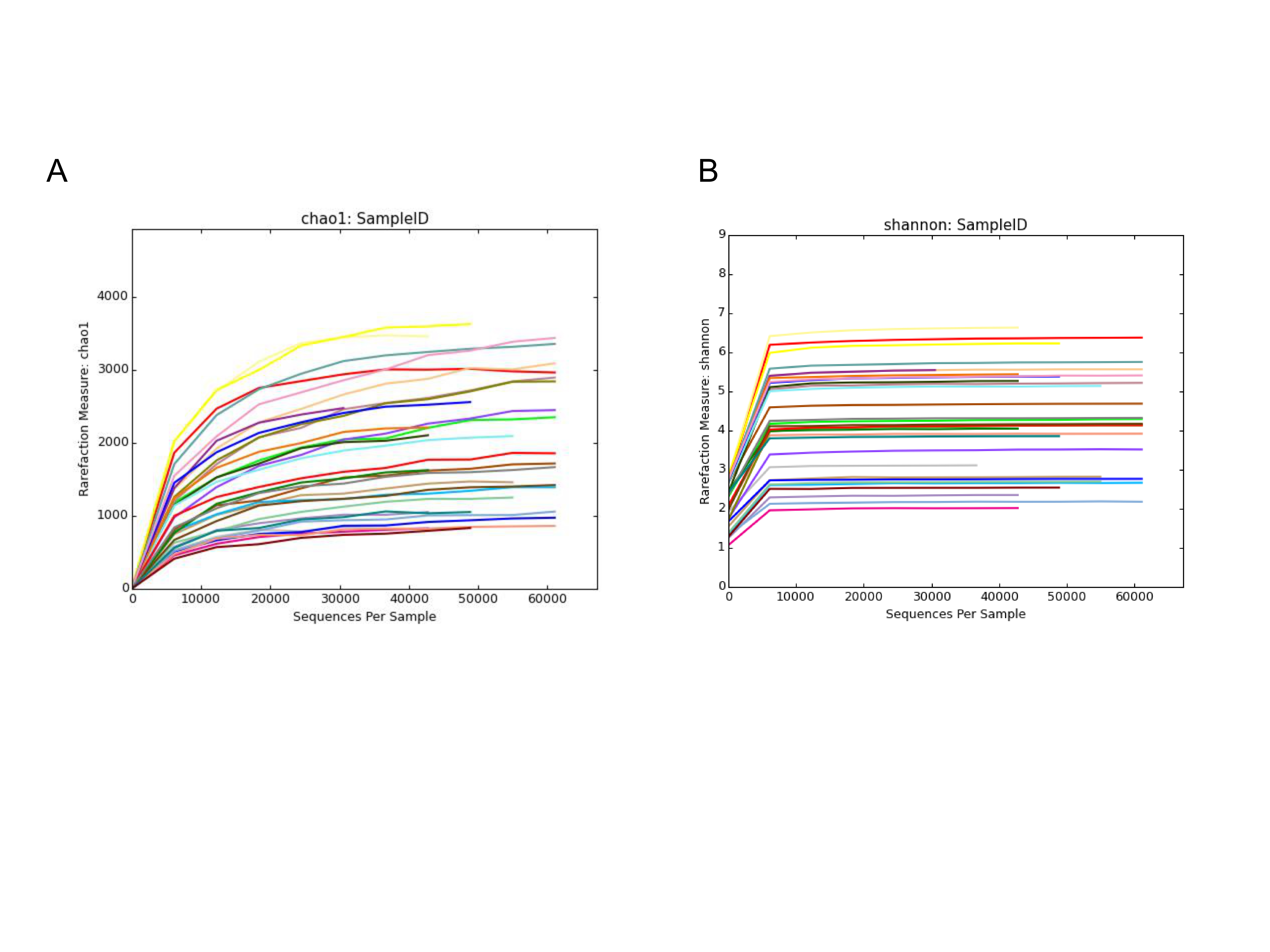

Supplement: Supplementary file 1 — Supplementary Material [file 41598_2019_42514_MOESM1_ESM.doc]
